# Evaluation of a point-of-care immunochromatographic assay for enteric fever in Dhaka, Bangladesh: a prospective diagnostic accuracy study

**DOI:** 10.1016/j.lanmic.2024.100983

**Published:** 2025-03

**Authors:** Sira J Munira, Nahidul Islam, Nowshin T Prithe, Anik Sarkar, Javan Esfandiari, Dhammika Gunasekera, Shuborno Islam, Tanjila Akter, Denise O Garrett, Samir K Saha, Jason R Andrews, Senjuti Saha, Richelle C Charles

**Affiliations:** a Child Health Research Foundation, Dhaka, Bangladesh; b Chembio Diagnostics Systems, Medford, NY USA; c Sabin Vaccine Institute, Washington, DC, USA; d Division of Infectious Diseases and Geographic Medicine, Stanford University School of Medicine, Stanford, CA, USA; e Massachusetts General Hospital, Harvard Medical School, Harvard T.H. Chan School of Public Health, Boston, MA, USA

## Abstract

**Background:**

There is a shortage of rapid, accurate, and low-cost assays for diagnosing enteric fever. The dual-path platform for typhoid (DPPT) assay had high accuracy in retrospective studies with banked plasma samples. We aimed to evaluate the diagnostic accuracy of the DPPT assay in a prospective study using fingerstick capillary blood.

**Methods:**

For this prospective diagnostic accuracy study, we enrolled children younger than 18 years, who presented at the Bangladesh Shishu Hospital and Institute (Dhaka, Bangladesh) with at least 3 days of fever. We collected blood and nasal swabs, and did the following assays to assess the accuracy of DPPT: blood culture, serological assays for typhoid fever (DPPT assay, Widal, and Test-it), and molecular assays to identify alternative aetiologies (eg, respiratory syncytial virus [RSV], influenza, dengue virus, and *Rickettsia* spp). The primary outcomes of the study were the sensitivity and specificity of the DPPT assay for diagnosis of enteric fever. We evaluated the accuracy of combined anti-haemolysin E and anti-lipopolysaccharide IgA readings using the DPPT assay in distinguishing culture-confirmed enteric fever from alternative aetiologies using receiver operating characteristic area under the curve (AUC). Given the imperfect reference standard diagnostics for enteric fever, we used Bayesian latent class models, incorporating results from the typhoid and alternative aetiology diagnostics, to estimate the sensitivity and specificity of the DPPT assay. We also did subgroup analyses for the assay across multiple biological variables.

**Findings:**

Between Aug 17, 2021, and July 16, 2022, we enrolled 501 participants who presented with at least 3 days of fever. 223 (45%) of 501 participants were female and 278 (55%) were male. 77 participants had culture-confirmed enteric fever (62 *Salmonella enterica* serotype Typhi and 15 *Salmonella enterica* serotype Paratyphi A) and 70 were culture-negative with PCR-confirmed alternative aetiology (34 influenza A or B, 22 dengue virus, seven *Rickettsia* spp, six RSV, and one participant co-infected with RSV and dengue virus). The AUC for DPPT on fingerstick capillary blood in distinguishing typhoid from alternative aetiologies was 0·969 (95% CI 0·943–0·994). In latent class analysis, the sensitivity of DPPT was 93% (95% credible interval [CrI] 87–97) and specificity was 89% (85–93). The balanced accuracy was higher for DPPT (91%, 95% CrI 87–94) than blood culture (81%, 78–85), Test-it (77%, 74–79), or Widal (70%, 67–73). Assay performance did not vary by sex, age, duration of fever at presentation, antibiotic use before presentation, *Salmonella* serotype, or sample type.

**Interpretation:**

The point-of-care DPPT assay achieved high diagnostic accuracy for enteric fever in a highly endemic community. This assay has the potential to improve clinical outcomes for enteric fever, allowing rapid diagnosis and treatment, and could facilitate more appropriate antimicrobial use.

**Funding:**

National Institute of Health, Bill & Melinda Gates Foundation, and Child Health Research Foundation.

## Introduction

Enteric fever, caused by *Salmonella enterica* serotype Typhi (*S* Typhi) and *Salmonella enterica* serotype Paratyphi A (*S* Paratyphi A), is responsible for an estimated 14 million illnesses and 135 000 deaths annually. The highest incidence of new cases and deaths occurs in southeast Asia and sub-Saharan Africa, where health systems are resource-limited and diagnostic capacity is restricted.^1^ In Bangladesh, there is a high incidence of typhoid (913 cases per 100 000 person-years) and paratyphoid (128 cases per 100 000 person-years) fever,^2^ which exceeds the threshold for high burden^3^^,^^4^ (100 cases per 100 000 person-years), and the burden is highest among children.^2^ No optimal rapid, accurate, and cost-effective assay is available for diagnosing patients with acute enteric fever.^5^, ^6^, ^7^

**Research in context**

**Evidence before this study**

We reviewed the evidence for rapid diagnostic tests for enteric fever by searching PubMed, medRxiv, and bioRxiv using the terms “enteric fever” OR “typhoid fever” OR “paratyphoid fever” OR “*Salmonella* Typhi” OR "*Salmonella* Paratyphi" AND “diagnostic” OR “rapid diagnostic” OR “point of care”, from database inception to Dec 31, 2023, with no language restrictions. A major difficulty in evaluating the accuracy of enteric fever diagnostics is the inadequate sensitivity of blood culture (around 60%), the most specific and widely used reference standard. Additionally, previous studies of enteric fever diagnostics have excluded *Salmonella enterica* serotype Paratyphi A (*S* Paratyphi A) and have not assessed how other biological variables (eg, age and duration of fever) affected the test’s performance. Several previous studies of commercially available rapid diagnostic tests for enteric fever showed moderate accuracy. A Cochrane systematic review of enteric fever rapid diagnostic tests found that none achieved balanced accuracy above 85%. Several studies have identified plasma IgA responses to haemolysin E and *Salmonella enterica* serotype Typhi (*S* Typhi) lipopolysaccharide as conferring high diagnostic accuracy, but none of these studies were done prospectively or using fingerstick capillary blood.

**Added value of this study**

We prospectively evaluated the dual-path platform for typhoid (DPPT) assay, using fingerstick capillary blood, among children presenting with acute febrile illness in a highly typhoid-endemic population. We included multiple febrile diagnostic assays alongside blood culture and used Bayesian latent class models to estimate the sensitivity and specificity of the DPPT assay. With this approach, we were able to generate a robust estimate of the diagnostic accuracy of the DPPT assay in a cohort of 501 febrile children in Dhaka, Bangladesh, with assessment of the influence of several biological parameters, including age, fever duration, and sample type. The assay displayed sensitivity (93%) and specificity (89%) that exceeded those of commercially available rapid diagnostic tests for enteric fever.

**Implications of all the available evidence**

Due to the lack of reliable rapid diagnostic tests, many patients with suspected enteric fever are managed on the basis of clinical signs and symptoms, which are not reliably distinguishable clinically from other febrile illnesses. This approach often results in excessive use of antibiotics, which might contribute to rising antimicrobial resistance. The DPPT assay showed high diagnostic accuracy for fingerstick capillary blood among children in a typhoid-endemic setting. Future studies should evaluate whether this assay can improve targeting of antibiotic use and facilitate disease burden estimates in areas lacking the capacity for blood culture surveillance.

The reference standard for enteric fever diagnosis is blood culture, but this method takes 2–4 days for results, has around 60% sensitivity, is affected by antibiotic use before blood culture, and is unavailable in many resource-constrained environments.^8^ The Widal test, the most commonly used rapid serological diagnostic method, has restricted sensitivity and specificity in areas endemic for typhoid.^9^ Although newer-generation serological diagnostics (eg, Tubex TF [IDL Biotech; Stockholm, Sweden], Typhidot Rapid IgG/IgM combo test [Reszon Diagnostics International; Selangor, Malaysia], and Enterocheck WB [Tulip Diagnostics; Goa, India]) have surpassed the Widal test in accuracy, the top-performing commercial assays still are suboptimal (ie, do not achieve a balanced accuracy above 85%).^10^ Therefore, many patients with suspected enteric fever are managed based on clinical assessments, which is unreliable as typhoid is difficult to distinguish clinically from other febrile illnesses.^10^^,^^11^ Dependence on clinical judgement alone has resulted in overdiagnosis of enteric fever and excessive use of antibiotics, contributing to antimicrobial resistance. A more accurate, rapid typhoid diagnostic test might improve clinical management and treatment of enteric fever, allow for more appropriate antimicrobial use, and facilitate disease burden estimates in areas lacking the capacity for blood culture surveillance.^11^, ^12^, ^13^

We have previously found that IgA antibody responses to haemolysin E and *S* Typhi lipopolysaccharide antigens can discriminate individuals with acute typhoid and paratyphoid fever from healthy or other febrile individuals in endemic areas in Asia and Africa.^14^^,^^15^ We adapted a point-of-care immunochromatographic dual-path platform technology (DPP; Chembio; Medford, NY USA) to detect anti-haemolysin E and anti-lipopolysaccharide IgA (dual-path platform for typhoid [DPPT] assay).^16^ This assay can be done with 10 μl of serum, plasma, venous blood, or capillary fingerprick blood and provides qualitative and quantitative results in 15–20 min using a microreader. The estimated cost is US$2 per test cassette and a one-time cost of $250 for the microreader.

Many commercial rapid diagnostic tests for enteric fever are based on detecting anti-IgM or IgG antibodies to *S* Typhi lipopolysaccharide O9 antigen (eg, Test-it Typhoid IgM [Life Assay; Cape Town, South Africa], Tubex TF, and Enterocheck WB). However, we believe the DPPT assay improves upon these existing assays for two reasons. First, we have previously found that the discriminatory value of anti-lipopolysaccharide IgA antibodies in identifying individuals with acute enteric fever in endemic areas was greater than IgM or IgG due to reduced cross-reactivity and longevity of response, respectively.^15^ Second, using a microreader provides the ability to set a quantitative cutoff of positivity that is not reliant on user interpretation.

Using a small, archived plasma set from Nepalese and Bangladeshi patients, we previously showed that the DPPT assay could discriminate enteric fever cases from febrile controls with a sensitivity of 90% and a specificity of 96%.^16^ However, further work was needed to assess the test performance in a larger cohort of patients and across differing age groups and sample types. In this study, we evaluated the accuracy of the DPPT assay in a cohort of children being evaluated for suspected enteric fever, assessing the effect of several biological parameters, including age, fever duration, and sample type.

## Methods

### Study design and participants

**Figure 1:**
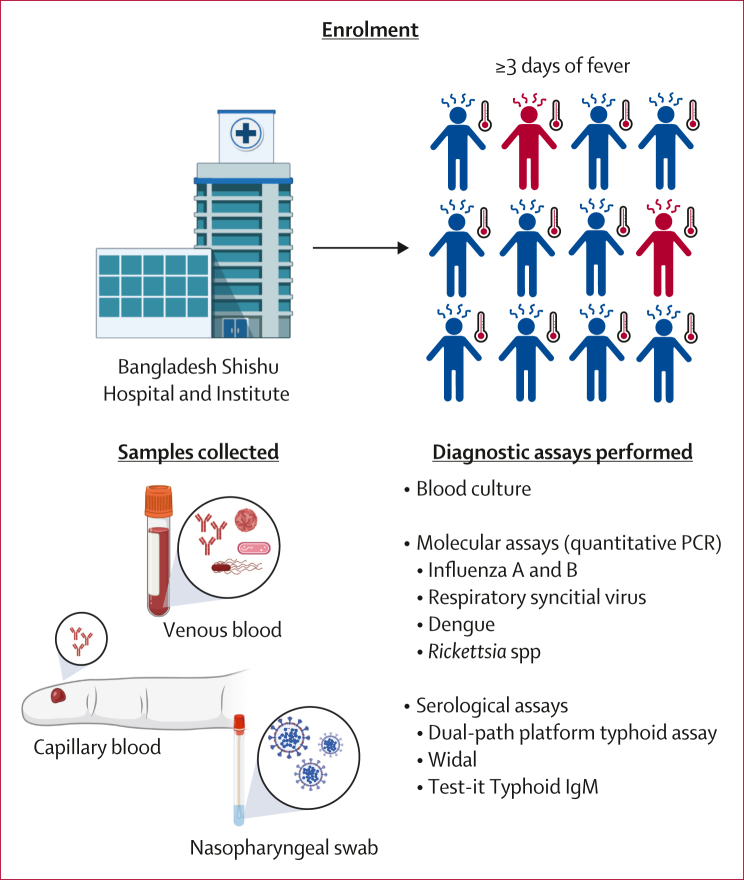
Study design

We did a prospective, diagnostic accuracy study, leveraging an ongoing prospective surveillance programme known as the Surveillance for Enteric Fever in Asia Project (SEAP) in a tertiary paediatric hospital (Bangladesh Shishu Hospital and Institute, previously known as Dhaka Shishu Hospital) in Dhaka, Bangladesh (figure 1). Patients who visited the outpatient department, were aged up to 18 years with a fever of at least 3 days duration within the past 7 days, resided within the hospital’s catchment area,^17^^,^^18^ and agreed to undergo blood culture were eligible for enrolment. Written informed consent was obtained from all participants’ parents or guardians. Informed written assent was also obtained from participants aged 16 years to younger than 18 years. Sex data was collected through self-report, with participants given the options of male and female. Race and ethnicity data were not collected. Patients who did not meet the inclusion criteria or agree to participate were excluded from the study. All research was approved by the Institutional Review Board for Human Subject Research or Ethical Review Committees of Bangladesh Institute of Child Health (BCH-ERC-01-10-2021) and Mass General Brigham (2019P000152).

### Procedures

The following information was collected from the patient or patient’s accompanying parent or guardian using a pretested semi-structured questionnaire: demographics, fever duration (number of days of fever before presentation), use of antibiotics for this febrile illness before presenting to the health-care facility, diagnosis of typhoid fever within the past 12 months, and any history of typhoid vaccination. Venous blood, capillary blood by fingerprick, and nasopharyngeal swabs were collected from participants. Plasma was isolated from venous blood and stored at –80°C until testing. Blood cultures were done using the BACTEC FX automated culture system (Becton Dickinson; Franklin Lakes, NJ, USA) and incubated at 37°C for 5 days. Positive bottles were subcultured onto sheep blood agar, chocolate agar, or MacConkey agar (Oxoid; Thermo Fisher Scientific; Hampshire, UK). *S* Typhi and *S* Paratyphi isolates were confirmed using standard biochemical assays along with O and H antisera (MAST ASSURE; Mast Group; Liverpool, UK).^2^

We used the DPPT assay to detect anti-haemolysin E and anti-lipopolysaccharide IgA from 10 μl of plasma, venous blood, or capillary blood, as previously described.^16^ This immunochromatographic assay consists of a test cassette and a portable, battery-powered microreader. The test cassette is designed with a sample path and reagent path, which converge in an analyte detection area that contains immobilised lipopolysaccharide, haemolysin E, and a control antigen (protein A). The intensity of each line was measured and quantified by the DPP Microreader II (Chembio). The Widal slide agglutination test (Human Diagnostics Worldwide; Wiesbaden, Germany) and Life Assay Test-it Typhoid IgM were done using plasma per the manufacturer’s instructions. A Widal titre of 1:160 or greater was considered positive. The Test-it Typhoid assay is a simple lateral flow assay that was considered positive if the control and test lines were visible.

DNA and RNA extractions were done on whole blood using the Quick-DNA/RNA MiniPrep Plus kit (Zymo Research; Irvine, CA, USA), and RNA was extracted from nasopharyngeal swabs using the Quick-DNA/RNA Viral MagBead kit (Zymo Research) following the manufacturer's protocol. The isolated whole blood RNA was used with the Luna Universal One-Step RT-qPCR Kit (New England BioLab; Ipswich, MA, USA) to detect dengue virus by quantitative PCR (qPCR) using previously published procedures.^19^ The isolated whole blood DNA was used with PerfeCTa qPCR ToughMix, Low ROX (Quantabio; Beverly, MA, USA) master mix to detect *Rickettsia* organisms by qPCR for the following targets, as previously described:^20^^,^^21^ 17 kDa (*Rickettsia* spp), 47 kDa (*Orientia tsutsugamushi*, the cause of scrub typhus), and ompB (run only on 17 kDa positive samples for *Ricketssia typhi*, the cause of murine typhus). Lastly, RT-qPCR was used to detect respiratory syncytial virus (RSV), influenza A, and influenza B from RNA extracted from nasopharyngeal swabs using AgPath-ID One-StepRT-qPCR kit (Thermo Fisher Scientific; Waltham, MA, USA). Specific primers, probes, and amplification conditions validated by the US Centers for Disease Control and Prevention were used for influenza A and influenza B;^22^^,^^23^ RSV primers and probes were used as previously described.^24^

### Outcomes

The primary outcomes of the study were the sensitivity and specificity of the DPPT assay for diagnosis of enteric fever. In the first set of analyses, sensitivity was estimated using blood culture-confirmed *S* Typhi or *S* Paratyphi A to define cases, and specificity was estimated with two different reference standards—all individuals with negative blood cultures and those with a confirmed alternative cause. Subsequently, Bayesian latent class models were used to estimate sensitivity and specificity among all participants.

Secondary outcomes were the association of anti-lipopolysaccharide and anti-haemolysin E IgA measurements using plasma, capillary whole blood, and venous whole blood and the effect of sex, age (<5 years *vs* ≥5 years), antibiotic use before presentation, duration of fever (<5 days *vs* ≥5 days), and *Salmonella* serotype on performance of the assay.

### Statistical analysis

We estimated sensitivity and specificity against the blood culture and alternative cause reference standards along with exact binomial 95% CIs. As the sensitivity of blood culture is around 60%, we then used Bayesian latent class models to estimate the sensitivity and specificity of the typhoid diagnostics along with the prevalence of enteric fever in the study population. This approach leverages previous information about the accuracy of blood culture and the other typhoid diagnostics (Test-it and Widal) and the joint results of all the assays to generate estimates for sensitivity and specificity. We used beta distributions for sensitivity and specificity with priors for blood culture from a recent meta-analysis^8^ (sensitivity 59%, 95% CI 54–64; specificity 99%, 99–100) and for Test-it (sensitivity 62%, 56–68; specificity 99%, 99–100) and Widal (sensitivity 49%, 43–55; specificity 84%, 78–88) from a recently published study.^10^ Following previously published methods,^10^ we used 200 000 Monte Carlo iterations, discarding the first 100 000, thinning by 100, and then reporting the median and 95% credible interval (CrI) for the resulting posterior distribution. We tested for convergence by Geweke’s diagnostic. For alternative etiologies, we used moderately informative priors, assuming that a positive test result by any of the alternative aetiology assays had 90–100% specificity for identifying individuals without typhoid; in sensitivity analyses, we used beta distributions of (1,1) and the estimates did not change. For DPPT assays, we identified a positivity threshold for the combination of lipopolysaccharide and haemolysin E that yielded 90% sensitivity for blood culture-confirmed cases and did latent class modelling using the resulting dichotomous classification. We used a threshold of 90% sensitivity based on the published target product profile for next-generation typhoid diagnostics.^13^ We used beta distributions of (1,1) for priors on the prevalence, sensitivity, and specificity of the DPPT assay. We also calculated balanced accuracy, defined as the average of sensitivity and specificity.

We also assessed the accuracy of the DPPT assay by receiver operator characteristic area under the curve (ROC AUC) using lipopolysaccharide IgA, HlyE IgA, or both combined. This analysis was done with all participants and separately with only those who had a confirmed aetiology.

We calculated sample size by simulation using latent class models. We anticipated 20% prevalence of enteric fever among participants based on previous studies in this setting. Enrolment of 500 participants was anticipated to achieve a CrI width on the sensitivity estimate of plus or minus 6%.

The association among capillary, venous, and plasma for DPPT measurements was assessed by Pearson’s correlation coefficient. The accuracy of the DPPT assay across multiple biological parameters (eg, sex, age [<5 years *vs* ≥5 years], antibiotic use before presentation, duration of fever [<5 days *vs* ≥5 days], *Salmonella* serotype) was assessed by the ROC AUC. DeLong’s test was used for comparing AUCs, and the threshold for significance was set at p<0·05.

All analyses were done using R version 4.3.0 and STATA version 14.

### Role of the funding source

The funders of the study had no role in study design, data collection, data analysis, data interpretation, or writing of the report.

## Results

**Figure 2:**
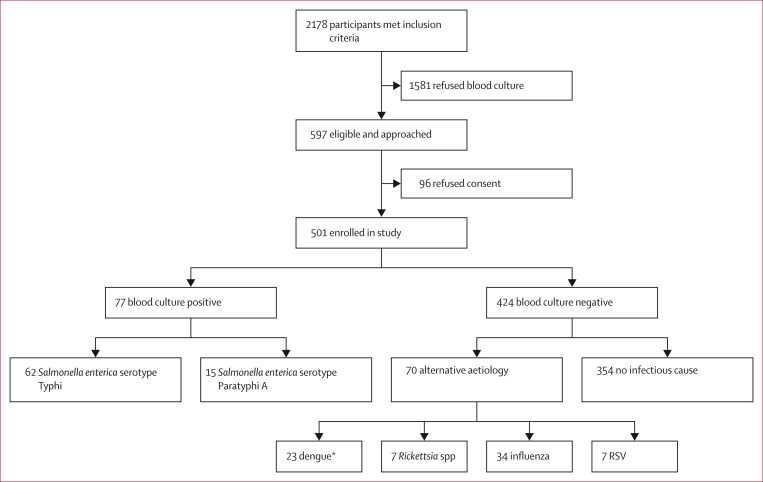
Study flowchart RSV=respiratory syncytial virus. ∗One patient was co-infected with RSV and dengue.

Between Aug 17, 2021, and July 16, 2022, we enrolled 501 participants who presented with at least 3 days of fever (figure 2). Most participants were under the age of 10 years (median age 4 years, IQR 2–7), 223 (45%) of 501 were female, 256 (51%) had taken antibiotics before presentation, and the median duration of fever at presentation was 5 days (IQR 4–7; table 1). We collected venous blood, capillary blood, and nasopharyngeal swabs from 501 (100%) participants, 299 (60%) participants, and 416 (83%) participants, respectively. 77 participants had culture-confirmed enteric fever (62 *S* Typhi and 15 *S* Paratyphi A) and 70 were culture-negative with PCR-confirmed alternative aetiology (34 influenza A or B, 22 dengue virus, seven *Rickettsia* spp, six RSV, and one participant co-infected with RSV and dengue virus; figure 2). One individual with *S* Typhi and one individual with *S* Paratyphi A were co-infected with dengue virus; these two participants were classified as having culture-positive enteric fever. The subcohorts of participants with enteric fever and confirmed alternative aetiology had similar characteristics (table 1; appendix 2 p 2).

**Table 1:** Characteristics of study participants

	All participants (n=501)	Enteric fever (n=77)	Alternativeaetiology (n=70)
Sex			
Female	223 (45%)	34 (44%)	33 (47%)
Male	278 (55%)	43 (56%)	37 (53%)
Age, years	4 (2–7)	5 (3–8)	5 (3–7)
<5	251 (50%)	35 (45%)	35 (50%)
5–9	181 (36%)	29 (38%)	23 (33%)
≥10	69 (14%)	13 (17%)	12 (17%)
Antibiotic use before presentation	256 (51%)	49 (64%)	25 (36%)
History of enteric fever	22 (4%)	2 (3%)	5 (7%)
Fever duration at presentation	5 (4–7)	5 (4–7)	4 (3–6)

Data are n (%) or median (IQR).

**Figure 3:**
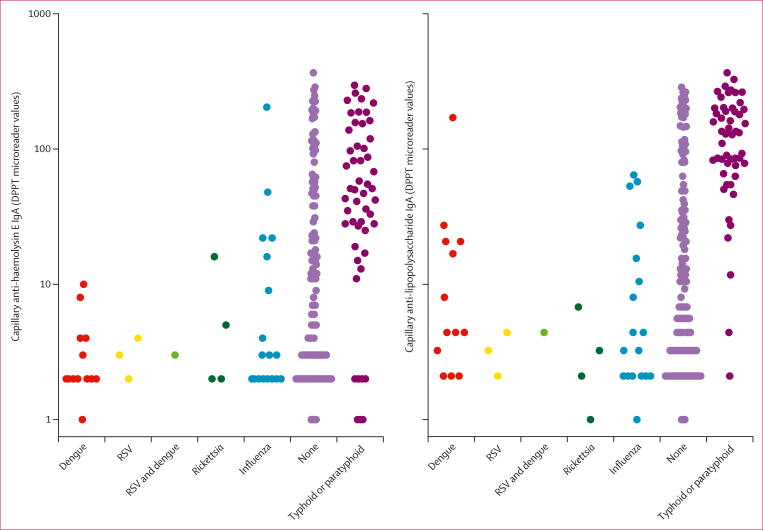
Anti-lipopolysaccharide and anti-haemolysin E IgA values measured by DPPT assay according to putative etiology of febrile illness Individuals with typhoid or paratyphoid and another aetiology (n=2) were depicted in the typhoid or paratyphoid group. RSV=respiratory syncytial virus. DPPT=dual-path platform for typhoid.

The distribution of anti-lipopolyssacharide and anti-haemolysin E IgA response measured by DPPT assay is plotted by diagnosis in figure 3. Using Youden’s optimal threshold, DPPT had a sensitivity of 95% (95% CI 82–99), much higher compared to Widal (52%, 48–56) and Test-it (31%, 28–37). The specificity of DPPT among alternative aetiology controls was 90% (95% CI 69–96) and among culture-negative controls was 80% (70–84). The Widal test had 94% (91–98) specificity among alternative aetiology controls and 91% (89–94) specificity among culture-negative controls, and the Test-it assays had 100% (100–100) specificity and 96% (94–98) specificity in these groups, respectively (appendix 2 p 3). The DPPT assay was considered positive if either lipopolysaccharide IgA or haemolysin E IgA line was positive; however, the AUC for lipopolysaccharide IgA is higher than for haemolysin E IgA. Using confirmed alternative aetiology as the control, the ROC AUC for lipopolysaccharide IgA was 0·953 (95% CI 0·920–0·986), for haemolysin E IgA was 0·854 (0·785–0·923), and combined was 0·969 (0·943–0·994; appendix 2 p 4). Among all participants, the AUC for lipopolyssacharide IgA was 0·953 (0·920–0·986), for haemolysin E IgA was 0·804 (0·741–0·867), and combined was 0·920 (0·897–0·944; appendix 2 p 5).

In the latent class model, the estimated prevalence of enteric fever was 24% (95% CrI 20–28). The DPPT assay had a sensitivity of 93% (87–97) and specificity of 89% (85–93). Blood culture and Test-it had higher specificity (100% for both, 95% CrI 100–100) but much lower sensitivity (blood culture 62%, 95% CrI 55–69 and Test-it 54%, 49–59). Balanced accuracy was higher for DPPT (91%, 95% CrI 87–94) than for blood culture (81%, 78–85), Test-it (77%, 74–79), or Widal (70%, 67–73; table 2).

**Table 2:** Accuracy of enteric fever diagnostics in a latent class model

	Sensitivity (95% CrI)	Specificity (95% CrI)	Balanced accuracy (95% CrI)
DPPT	93% (87–97)	89% (85–93)	91% (87–94)
Test-it	54% (49–59)	100% (100–100)	77% (74–79)
Widal	48% (43–53)	92% (90–94)	70% (67–73)
Blood culture	62% (55–69)	100% (100–100)	81% (78–85)

CrI=credible interval. DPPT=dual-path platform for typhoid.

**Figure 4:**
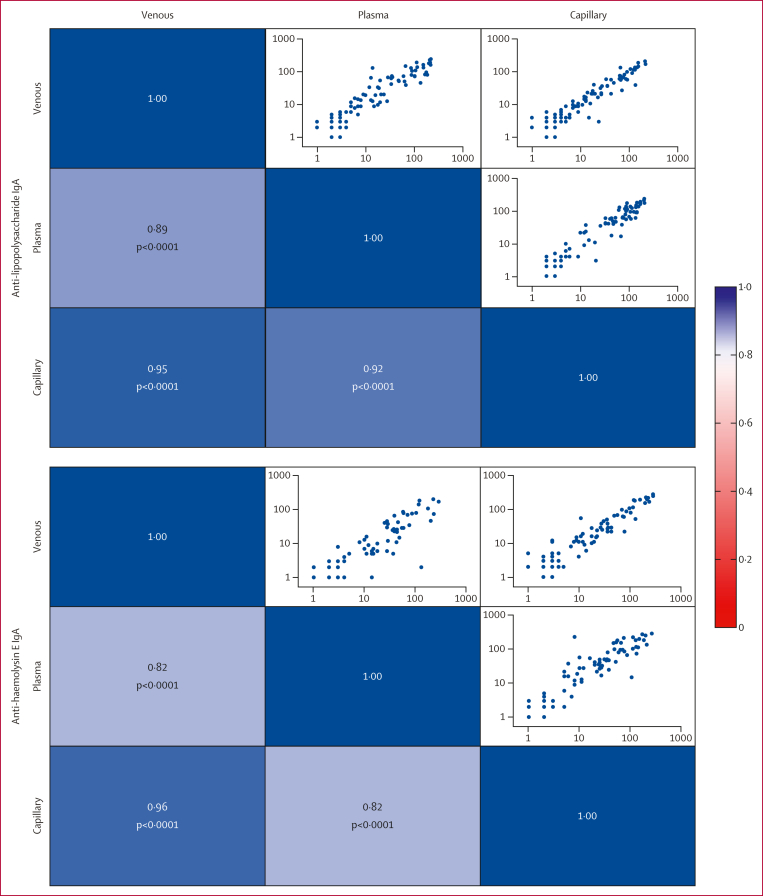
Correlation of DPPT quantitative values by sample type Pearson’s correlation r values and plot of DPPT anti-lipopolysaccharide and anti-haemolysin E IgA microreader values using plasma, capillary whole blood, and venous whole blood. DPPT=dual-path platform for typhoid.

We assessed the robustness of DPPT by evaluating the ROC AUC with a 95% CI across various biological parameters (table 3). Using confirmed alternative aetiology as our negative control, we found that there were no significant differences in the performance of the assay by sex (female AUC 0·97, 95% CI 0·93–1·00 *vs* male 0·96, 0·93–0·99; p=0·81), age (<5 years 0·97, 0·94–1·00 *vs* ≥5 years 0·97, 0·93–1·00; p=0·95), duration of fever at presentation (<5 days 0·96, 0·91–1·00 *vs* ≥5 days 0·98, 0·95–1·00; p=0·42), antibiotic use before presentation (yes 0·98, 0·96–1·00 *vs* no 0·95, 0·90–0·99; p=0·19), or infecting typhoidal serovar (*S* Typhi 0·98, 0·96–1·00 *vs S* Paratyphi A 0·97, 0·94–0·99; p=0·40). We also assessed performance across sample types and found that anti-lipopolysaccharide and anti-haemolysin E antibody measurements were highly correlated across plasma, capillary whole blood, and venous whole blood (figure 4).

**Table 3:** Performance of the dual-path platform for typhoid assay in subgroups

	Area under the curve (95% CI)	p value
Sex		0·81
Female	0·97 (0·93–1·00)	
Male	0·96 (0·93–0·99)	
Age, years		0·95
<5	0·97 (0·94–1·00)	
≥5	0·97 (0·93–1·00)	
Duration of fever, days		0·42
<5	0·96 (0·91–1·00)	
≥5	0·98 (0·95–1·00)	
Antibiotic use before presentation		0·19
Yes	0·98 (0·96–1·00)	
No	0·95 (0·90–0·99)	
*Salmonella* enterica serotype		0·40
Typhi	0·98 (0·96–1·00)	
Paratyphi A	0·97 (0·94–0·99)

## Discussion

There is a crucial need for a reliable rapid diagnostic test for enteric fever to guide treatment in low-income and middle-income countries, where enteric fever is a major cause of morbidity and mortality. In the absence of such a test, many patients receive antibiotics unnecessarily, as enteric fever is difficult to distinguish clinically from other common febrile illnesses. In this prospective study in a typhoid-endemic setting, using blood culture, other typhoid rapid diagnostic tests, and molecular diagnostics for other common pathogens, we found that the DPPT assay using fingerstick capillary blood achieved 93% sensitivity and 89% specificity for enteric fever. These results were robust to sex, age, duration of fever at presentation, and antibiotic use before presentation, and for both typhoid and paratyphoid diagnosis. The performance of the DPPT assay surpassed that of other commercially available assays for enteric fever as reported in previous studies.^6^^,^^10^ With an estimated sensitivity of 90%, the DPPT assay could in principle increase detection of enteric fever cases by 50% compared with blood culture, which has been estimated to have around 60% sensitivity.^8^

The DPPT assay builds upon a body of evidence showing that measurement of anti-lipopolysaccharide (O9 antigen) and anti-haemolysin E IgA in blood samples can accurately classify individuals with acute *S* Typhi and *S* Paratyphi A infection.^15^^,^^25^, ^26^, ^27^ A previous retrospective study showed that the DPPT assay has excellent quantitative correlation with anti-haemolysin E and anti-lipopolysaccharide IgA responses, as measured by kinetic ELISA, but, to our knowledge, our study was the first to prospectively evaluate the diagnostic accuracy of this assay.^16^ Although the inclusion of haemolysin E improves the discriminatory power of the assay, this increase in accuracy was small in this cohort compared with our previous findings.^15^^,^^16^ This difference might be explained by the fact that this current cohort was primarily under the age of ten years, and haemolysin E values at presentation have been shown to increase with age, whereas there are no age-related differences in lipopolysaccharide values at presentation.^14^^,^^15^ Further evaluation of the assay in adults is needed to determine whether haemolysin E adds value to the assay’s performance.

Since *S* Typhi and *S* Paratyphi A share haemolysin E and the O12 antigen on their lipopolysaccharide, the DPPT assay cannot discriminate between these two typhoidal serovars, and we found no differences in the performance of the assay when we did our analysis by *Salmonella* serotype. We also included a number of alternative aetiology controls (eg, dengue virus, *Rickettsia* spp*,* including *O tsutsugamushi*, influenza, and RSV) to ensure there was no cross-reactivity with other potential febrile illnesses with similar clinical presentation to enteric fever, which are also prevalent in Dhaka. We did not identify patients with other invasive bacteraemias in our cohort, but our previous analysis of lipopolysaccharide and haemolysin E measured by DPPT or ELISA showed little cross-creativity with other Gram-positive or Gram-negative bacteria.^15^^,^^16^

A limitation of our study is that our cohort only included children at a single site that was highly endemic for enteric fever. Although enteric fever mostly affects young children and adolescents, caution is required before generalising these results to adults and to other geographical areas for several reasons. First, we have previously shown that the antibody kinetics for lipopolysaccharide IgA and haemolysin E IgA differ by age group. Although lipopolysaccharide antibody levels at presentation do not differ by age, the antibody decay rate for both antigens decreases with age.^14^ We did not find any difference in accuracy in young children (<5 years) versus older children (≥5 years) in this analysis, but it will be important to study the performance of the DPPT assay in cohorts that include adults, as the specificity of the assay in older age groups might differ. Second, Dhaka is a highly endemic area for enteric fever; thus, individuals in Dhaka might have more robust and rapid antibody responses from previous exposure than in areas with a lower burden of infection. Third, invasive non-typhoidal *Salmonella* is not prevalent in Dhaka, so we could not assess the cross-reactivity of anti-lipopolysaccharide and anti-haemolysin E antibodies with this infection. We have previously shown that haemolysin E IgA and lipopolysaccharide IgA antibodies at presentation could distinguish typhoidal *Salmonella* from non-typhoidal *Salmonella*.^14^ However, further evaluation of the assay in areas where invasive non-typhoidal *Salmonella* is prevalent (eg, endemic areas in Africa) and in endemic areas with lower burdens of infection is warranted, as haemolysin E can rarely be found in other *Salmonella* serovars and invasive non-typhoidal *Salmonella* can share some components of lipopolysaccharide (eg, O12, O9) with *S* Typhi and *S* Paratyphi A. Finally, blood culture is not a reliable reference standard for enteric fever. We used Bayesian latent class modeling and included alternative aetiology controls to combat this. However, due to the poor sensitivity of blood culture, it is possible that our alternative aetiology controls could have been co-infected with *S* Typhi or *S* Paratyphi A, potentially affecting our specificity estimate. Furthermore, we did not perform stool culture, which has lower sensitivity than blood culture in enteric fever, and we are unable to assess the sensitivity of this assay for identifying gastrointestinal shedding.

Despite these limitations, our study shows that the DPPT assay can accurately discriminate enteric fever from other febrile aetiologies in a highly endemic area. This assay has the potential to fill a critical gap in enteric fever diagnostics and improve clinical management and treatment of enteric fever. Although the performance of the assay is encouraging, further work is needed to assess its performance in older age groups and diverse geographical locations with varying levels of enteric fever incidence.

## Data sharing

All code required to replicate study output is available at https://github.com/CHRF-Genomics/DPPT-RapidDiagnosticTest-EntericFever.

## Declaration of interests

JE and DG are currently employed at Chembio Diagnostics Systems (Medford, NY, USA). However, we purchased the kits from Chembio and they had no role in the study design or analysis. All other authors declare no competing interests.
