# Supplementary material for: Evaluation of a point-of-care immunochromatographic assay for enteric fever in Dhaka, Bangladesh: a prospective diagnostic accuracy study
Source: Lancet Microbe. 2025 Mar;6(3):None. doi: 10.1016/j.lanmic.2024.100983 (PMC11876101; doi:10.1016/j.lanmic.2024.100983)
Supplement: Supplementary appendix 1 [file mmc1.pdf]

THE LANCET
Microbe

Supplementary appendix 1

This translation in Bengali was submitted by the authors and we reproduce it as supplied. It has not been peer reviewed. *The Lancet*'s editorial processes have only been applied to the original in English, which should serve as reference for this manuscript.

'এই [বাংলায়] অনুবাদটি লেখকরা জমা দিয়েছিলেন এবং এটি যেমনভাবে দেওয়া হয়েছে আমরা সেইভাবেই পুনরায় বর্ণনা করছি। এটি কোনো সমকক্ষ ব্যক্তি পর্যালোচনা করেননি। দ্য ল্যানসেট-এর সম্পাদকীয় প্রক্রিয়াগুলি শুধুমাত্র মূল ইংরেজিতে প্রয়োগ করা হয়েছে, যা এই পান্ডুলিপির রেফারেন্স হিসাবে কাজ করবে।'

Supplement to: Munira SJ, Islam N, Prithe NT, et al. Evaluation of a point-of-care immunochromatographic assay for enteric fever in Dhaka, Bangladesh: a prospective diagnostic accuracy study. *Lancet Microbe* 2025. https://doi.org/10.1016/j.lanmic.2024.100983

Prospective evaluation of a point-of-care immunochromatographic assay for enteric fever in Dhaka, Bangladesh: a diagnostic accuracy study

Appendix-1
Bengali translation of the summary

পটভূমি

টাইফয়েড ও প্যারাটাইফয়েড জ্বর, সালমোনেলা টাইফি ও প্যারাটাইফি দ্বারা সৃষ্ট সংক্রামক রোগ। দ্রুত, নির্ভুল ও স্বল্প মূল্যে এই জ্বর শনাক্তের জন্য সহজলভ্য পদ্ধতির সংখ্যা অনেক সীমিত। ডুয়াল পাথ প্লাটর্ফম ফর টাইফয়েড (ডিপিপিটি) পরীক্ষা ব্যবহার করে পূর্ববর্তী গবেষণাতে নির্ভুলভাবে টাইফয়েডের জীবাণু চিহ্নিত করা সম্ভব হয়েছে। এই গবেষণায় একটি নির্দিষ্ট জনগোষ্ঠীর আঙুলের ক্যাপিলারী রক্ত পরীক্ষার মাধ্যমে ডিপিপিটি পদ্ধতির ডায়াগনস্টিক যথার্থতা মূল্যায়ন করা হয়।

পদ্ধতি

বাংলাদেশ শিশু হসপিটাল ও ইন্সটিটিউট এ আগত তিন দিন বা এর বেশি সময়ের জ্বরাক্রান্ত ১৮ বছরের কম বয়সী শিশুদের এই গবেষণায় অন্তর্ভুক্ত করা হয়। তাদের রক্তের নমুনা থেকে ব্লাড কালচার ও টাইফয়েড জ্বর নির্ণায়ক সেরোলজিকাল পরীক্ষা (ডিপিপিটি, উইডাল এবং টেস্ট-ইট) পরীক্ষা সম্পাদন করা হয়। এছাড়া বিভিন্ন মলিকুলার পরীক্ষার মাধ্যমে টাইফয়েড ব্যাতিত অন্যান্য সংক্রমণকারী অণুজীব যেমন- রেসপিরেটরি সিনসাইটিয়াল ভাইরাস (আরএসভি), ইনফ্লুয়েঞ্জা, ডেঙ্গু এবং রিকেটসিয়া ব্যাকটেরিয়ার উপস্থিতি রক্ত ও নাকের নমুনাতে নিশ্চিত করা হয়। এই গবেষণার প্রাথমিক ফলাফল হল টাইফয়েড ও প্যারাটাইফয়েড জ্বর সনাক্তের জন্য ডিপিপিটি পরীক্ষার সেনসিটিভি এবং স্পেসিফিটি নির্ধারণ করা। এই জ্বরকে অন্যান্য সংক্রমণকারী অণুজীব থেকে নির্ভুলভাবে সনাক্ত করার জন্য হেমোলাইসিন ই (anti-HlyE IgA) এবং লিপোপলিস্যাকারাইড (anti-LPS IgA) আইজি এ অ্যান্টিবডির সমন্বিত রিডিং ব্যবহার করে receiver operating characteristic (ROC) area under the curve (AUC) পরিমাপ করা হয়। টাইফয়েড এবং এই টেস্টগুলোর ফলাফলকে বায়েসিয়ান ল্যাটেন্ট (Bayesian latent) মডেলে অন্তর্ভুক্ত করে ডিপিপিটি পরীক্ষার সেনসিটিভি এবং স্পেসিফিটি নির্ণয় করা হয়। এছাড়া এই পরীক্ষার কার্যকারিতা নির্ধারণের জন্য বিভিন্ন ক্লিনিক্যাল বৈশিষ্ট্যের মধ্যে সাবগ্রুপ বিশ্লেষণ করা হয়।

ফলাফল

১৭ই আগস্ট, ২০২১ থেকে ১৬ই জুলাই, ২০২২ এর মধ্যে অংশগ্রহণকারী ৫০১ জন শিশুর মধ্যে ৬২ জন টাইফয়েড ও ১৫ জন প্যারাটাইফয়েড জ্বরে আক্রান্ত হিসেবে শনাক্ত করা যায়। এছাড়া অন্যান্য জীবাণু দিয়ে ৭০ জন (৩৪ জন ইনফ্লুয়েঞ্জা, ২২ জন ডেঙ্গু, ৭ জন রিকেটসিয়া, ৬ জন আরএসভি এবং ১ জন আরএসভি ও ডেঙ্গু দুটি দ্বারাই) শিশু আক্রান্ত ছিলো। ডিপিপিটি দিয়ে আঙুলের ক্যাপিলারী রক্তে টাইফয়েড শনাক্তকরনের জন্য AUC হিসাব করা হয়েছে ০.৯৬৯ (৯৫% কনফিডেন্স

Prospective evaluation of a point-of-care immunochromatographic assay for enteric fever in Dhaka, Bangladesh: a diagnostic accuracy study

Appendix-1
Bengali translation of the summary

ইন্টারভাল, ০.৯৪৩ - ০.৯৯৪)। ল্যাটেন্ট ক্লাস এন্যালাইসিস এর মাধ্যমে এই পরীক্ষার সেনসিটিভি ৯৩% (৯৫% ক্রেডিবল ইন্টারভাল, ৮৭-৯৭%) এবং স্পেসিফিটি ৮৯% (৯৫% ক্রেডিবল ইন্টারভাল, ৮৫-৯৩%) নির্ণয় করা হয়েছে। ব্লাড কালচার (৮১%; সেনসিটিভি, ৬২%; স্পেসিফিটি, ১০০%), টেস্ট-ইট (৭৭%; সেনসিটিভি, ৫৪%; স্পেসিফিটি ১০০%), এবং উইডাল টেস্ট (৭০%; সেনসিটিভি, ৪৮%; স্পেসিফিটি ৯২%) থেকে ডিপিপিটি পরীক্ষার (৯১%; সেনসিটিভি, ৯৩%; স্পেসিফিটি ৮৯%) ব্যালান্সড একিউরেসি বেশি পাওয়া গিয়েছে। রোগীর জেন্ডার, বয়স, জ্বরের সময়কাল, এই অসুস্থতার আগে অ্যান্টিবায়োটিকের ব্যবহার, টাইফয়েড জীবাণুর প্রকারভেদ বা রক্তের নমুনার ভিন্নতার কারনে এই পরীক্ষাটির কার্যকারিতায় কোন পরিবর্তন হয়নি।

ব্যাখ্যা

টাইফয়েড প্রবন এলাকায় এই জ্বর শনাক্তকরণে পয়েন্ট-অফ-কেয়ার ডিপিপিটি পরীক্ষাটি অধিকতর ডায়াগনস্টিক নির্ভুলতা প্রদর্শন করেছে। এর মাধ্যমে টাইফয়েড জ্বর দ্রুত ও সল্প খরচে নির্ণয় করে উপযুক্ত অ্যান্টিমাইক্রোবিয়াল ঔষধ ব্যবহার, সর্বোপরি এই রোগের ক্লিনিক্যাল ফলাফল উন্নত করার ও প্রার্দুরভাব কমানোর সম্ভাবনা রয়েছে।

ফান্ডিং

এই গবেষণাটি ন্যাশনাল ইনস্টিটিউট অফ হেলথ (R21 AI161770), বিল অ্যান্ড মেলিন্ডা গেটস ফাউন্ডেশন (INV-008335) এবং চাইল্ড হেলথ রিসার্চ ফাউন্ডেশন এর অর্থায়নে সম্পাদিত হয়েছে।
