# Supplementary material for: Evaluation of a point-of-care immunochromatographic assay for enteric fever in Dhaka, Bangladesh: a prospective diagnostic accuracy study
Source: Lancet Microbe. 2025 Mar;6(3):None. doi: 10.1016/j.lanmic.2024.100983 (PMC11876101; doi:10.1016/j.lanmic.2024.100983)
Supplement: Supplementary appendix 2 [file mmc2.pdf]

THE LANCET
Microbe

Supplementary appendix 2

This appendix formed part of the original submission and has been peer reviewed. We post it as supplied by the authors.

Supplement to: Munira SJ, Islam N, Prithe NT, et al. Evaluation of a point-of-care immunochromatographic assay for enteric fever in Dhaka, Bangladesh: a prospective diagnostic accuracy study. *Lancet Microbe* 2025. https://doi.org/10.1016/j.lanmic.2024.100983

Prospective evaluation of a point-of-care immunochromatographic assay for enteric fever in Dhaka, Bangladesh: a diagnostic accuracy study

Appendix-2

TABLE OF CONTENTS

No.	Title	Pages
Supplemental Table 1.	Diagnosis by age group.	Page 2
Supplemental Table 2.	Accuracy of the DPPT Assay, Widal, and Test-it without Latent class modeling.	Page 3
Supplemental Figure 1.	Receiver operating characteristic (ROC) area under the curve (AUC) for anti-LPS and HlyE IgA responses using the DPPT assay among confirmed etiologies.	Page 4
Supplemental Figure 2.	Receiver operating characteristic (ROC) area under the curve (AUC) for anti-LPS and HlyE IgA response using the DPPT assay all participants.	Page 5

Prospective evaluation of a point-of-care immunochromatographic assay for enteric fever in Dhaka, Bangladesh: a diagnostic accuracy study

Appendix-2

Supplemental Table 1. Diagnosis by age group.

Diagnosis	Age < 5 y n=251	Age 5 to 9 y n=181	Age ≥ 10 y n=69	Total
**Enteric fever Case, n**	35	29	13	77
Typhoid	30	22*	10	62
Paratyphoid	5	7*	3	15
**Alternative etiology controls, n**	35	23	12	70
Influenza (A or B)	21	9	4	34
RSV	3	3	0	6
Dengue virus	6	9	7	22
Dengue virus & RSV	0	1	0	1
Rickettsia spp.	5	1	1	7
**No confirmed etiologies, n**	181	129	44	354

*co-infected enteric fever case with dengue, considered a case only (not included in AE controls).

Prospective evaluation of a point-of-care immunochromatographic assay for enteric fever in Dhaka, Bangladesh: a diagnostic accuracy study

Appendix-2

Supplemental Table 2. Accuracy of the DPPT Assay, Widal, and Test-it without Latent class modeling

**Tests**		**Culture-negative controls**	**Alternative etiology controls**
	**Sensitivity (95% CI)**	**Specificity (95% CI)**	**Specificity (95% CI)**
DPPT assay*	95% (82-99)	80% (70-84)	90% (69-96)
Widal**	52% (48-56)	91% (89-94)	94% (91-98)
Test-it	31% (28-37)	96% (94-98)	100% (100-100)

*Using Youden's Optimal Threshold

** Widal positive if ≥ 1:160

Prospective evaluation of a point-of-care immunochromatographic assay for enteric fever in Dhaka, Bangladesh: a diagnostic accuracy study

Appendix-2

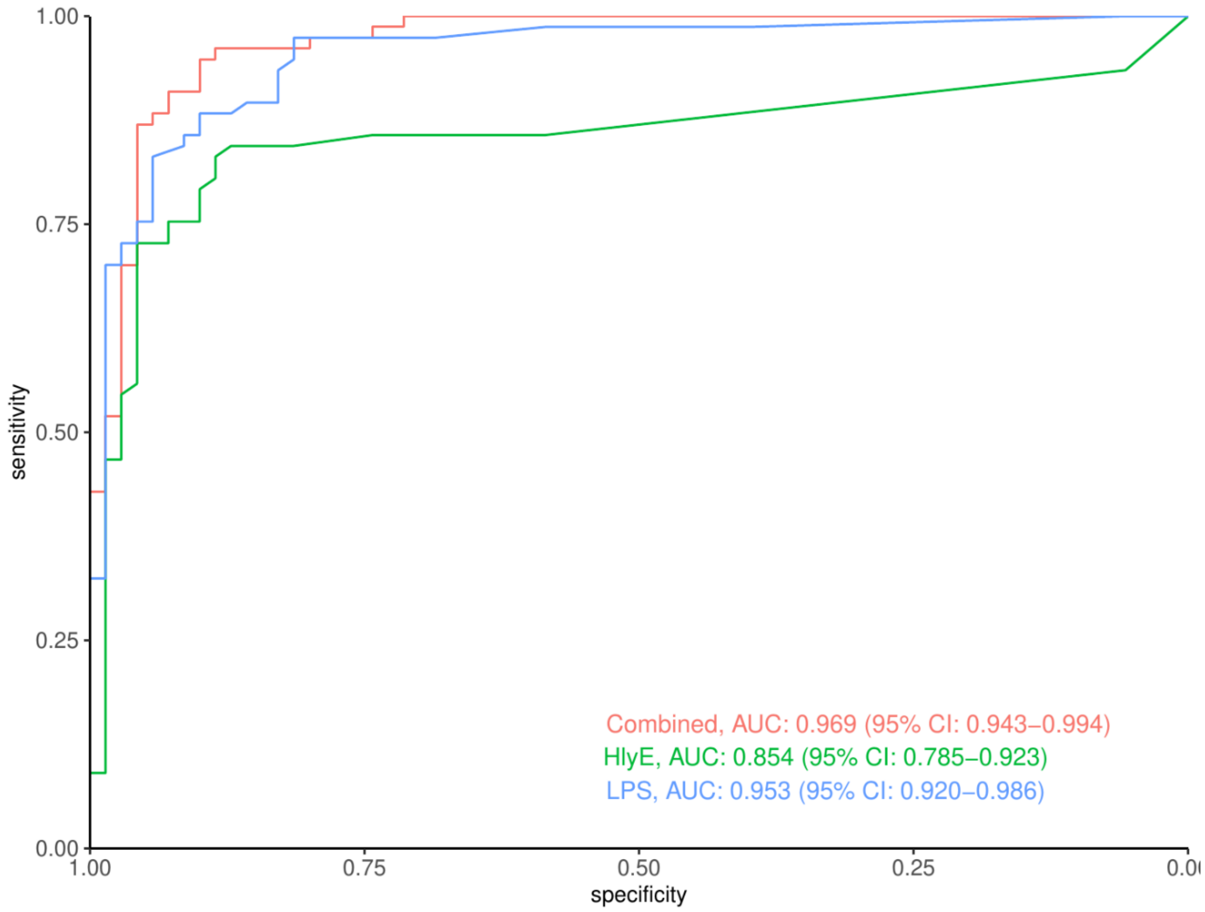

Supplemental Figure 1. Receiver operating characteristic (ROC) area under the curve (AUC) for anti-LPS and HlyE IgA response using the DPPT assay among confirmed etiologies. ROC is plotting specificity versus sensitivity. AUC is shown with 95% confidence interval (CI).

Prospective evaluation of a point-of-care immunochromatographic assay for enteric fever in Dhaka, Bangladesh: a diagnostic accuracy study

Appendix-2

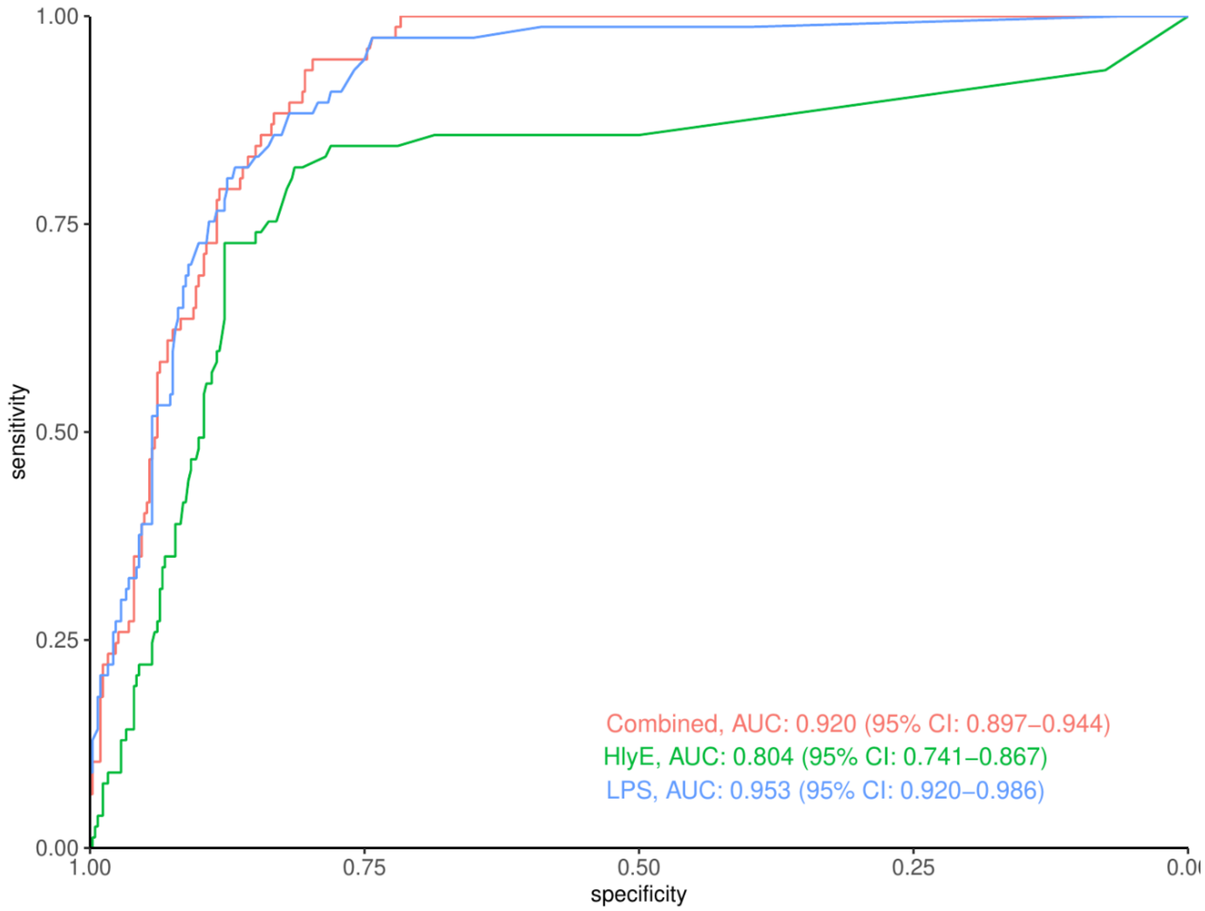

Supplemental Figure 2. Receiver operating characteristic (ROC) area under the curve (AUC) for anti-LPS and HlyE IgA response using the DPPT assay among all participants. ROC is plotting specificity versus sensitivity. AUC is shown with 95% confidence interval (CI).
